# Supplementary material for: Bayesian refinement of protein structures and ensembles against SAXS data using molecular dynamics
Source: PLoS Comput Biol. 2017 Oct 18;13(10):e1005800. doi: 10.1371/journal.pcbi.1005800 (PMC5662244; doi:10.1371/journal.pcbi.1005800)
Supplement: S1 Archive — (BZ2) [file pcbi.1005800.s011.tar.bz2 › gromacs_4.62_bayesian_saxs_refi/share/html/online/g_mindist.html]

g\_mindist


| g\_mindist Main Table of Contents |

**VERSION 4.6  
Sat 19 Jan 2013**

---

### Description

g\_mindist computes the distance between one group and a number of
other groups. Both the minimum distance
(between any pair of atoms from the respective groups)
and the number of contacts within a given
distance are written to two separate output files.
With the -group option a contact of an atom in another group
with multiple atoms in the first group is counted as one contact
instead of as multiple contacts.
With -or, minimum distances to each residue in the first
group are determined and plotted as a function of residue number.

With option -pi the minimum distance of a group to its
periodic image is plotted. This is useful for checking if a protein
has seen its periodic image during a simulation. Only one shift in
each direction is considered, giving a total of 26 shifts.
It also plots the maximum distance within the group and the lengths
of the three box vectors.

Other programs that calculate distances are g\_dist
and g\_bond.

### Files

| option | filename | type | description |
| --- | --- | --- | --- |
| **-f** | traj.xtc | Input | Trajectory: xtc trr trj gro g96 pdb cpt |
| **-s** | topol.tpr | Input, Opt. | Structure+mass(db): tpr tpb tpa gro g96 pdb |
| **-n** | index.ndx | Input, Opt. | Index file |
| **-od** | mindist.xvg | Output | xvgr/xmgr file |
| **-on** | numcont.xvg | Output, Opt. | xvgr/xmgr file |
| **-o** | atm-pair.out | Output, Opt. | Generic output file |
| **-ox** | mindist.xtc | Output, Opt. | Trajectory: xtc trr trj gro g96 pdb |
| **-or** | mindistres.xvg | Output, Opt. | xvgr/xmgr file |

### Other options

| option | type | default | description |
| --- | --- | --- | --- |
| **-[no]h** | bool | no | Print help info and quit || **-[no]version** | bool | no | Print version info and quit || **-nice** | int | 19 | Set the nicelevel || **-b** | time | 0 | First frame (ps) to read from trajectory || **-e** | time | 0 | Last frame (ps) to read from trajectory || **-dt** | time | 0 | Only use frame when t MOD dt = first time (ps) || **-tu** | enum | ps | Time unit: fs, ps, ns, us, ms or s || **-[no]w** | bool | no | View output .xvg, .xpm, .eps and .pdb files || **-xvg** | enum | xmgrace | xvg plot formatting: xmgrace, xmgr or none || **-[no]matrix** | bool | no | Calculate half a matrix of group-group distances || **-[no]max** | bool | no | Calculate \*maximum\* distance instead of minimum || **-d** | real | 0.6 | Distance for contacts || **-[no]group** | bool | no | Count contacts with multiple atoms in the first group as one || **-[no]pi** | bool | no | Calculate minimum distance with periodic images || **-[no]split** | bool | no | Split graph where time is zero || **-ng** | int | 1 | Number of secondary groups to compute distance to a central group || **-[no]pbc** | bool | yes | Take periodic boundary conditions into account || **-[no]respertime** | bool | no | When writing per-residue distances, write distance for each time point || **-[no]printresname** | bool | no | Write residue names |

---

http://www.gromacs.org  
gromacs@gromacs.org
